# Improving Treatment of Children with Autism Spectrum Disorder in Low- and Middle-Income Countries: The Role of Non-specialist Care Providers

**DOI:** 10.1371/journal.pmed.1001573

**Published:** 2013-12-17

**Authors:** Mashudat A. Bello-Mojeed, Muideen O. Bakare

**Affiliations:** 1 Child and Adolescent Centre, Federal Neuro-Psychiatric Hospital, Lagos, Nigeria; 2 Child and Adolescent Unit, Federal Neuro-Psychiatric Hospital, New Haven, Enugu, Nigeria

## Abstract

Mashudat Bello-Mojeed and Muideen O. Bakare discuss the unmet challenges in care of children with autism spectrum disorders in low- and middle-income countries.

*Please see later in the article for the Editors' Summary*

**Linked Research Article**

This Perspective discusses the following new study published in *PLOS Medicine*:

Reichow B, Servili C, Yasamy MT, Barbui C, Saxena S (2013) Non-specialist psychosocial intervention for children and adolescents with intellectual disability or lower-functioning autism spectrum disorders: a systematic review. PLoS Med 10(12): e1001572. doi:10.1371/journal.pmed.1001572

In a systematic review, Brian Reichow and colleagues assess the evidence that non-specialist care providers in community settings can provide effective interventions for children and adolescents with intellectual disabilities or lower-functioning autism spectrum disorders.

## Interventions for Children with Autism Spectrum Disorder: An Unmet Challenge

Developmental disabilities including autism spectrum disorder (ASD) and intellectual disability (ID) have been ranked among the leading mental health-related causes of the global burden of disease with lifelong effects on children under the age of 10 years [1]. With under-five child mortality declining in resource-poor countries [1], an increasing number of children will live on to experience an increasing burden of neurodevelopmental disorders (NDDs) while the family shares a huge burden of care-giving [2]. Diagnosis of ASD in children is reported to co-exist with other conditions such as intellectual disabilities, epilepsy, tuberous sclerosis, attention deficit hyperactivity disorder (ADHD), and emotional disorders [3]. Many of these children show deterioration in social skills, declines in cognitive functioning, delays in language skills, and poor motor skills.

In a systematic review published this week in *PLOS Medicine*, Brian Reichow and colleagues examine the benefits and adaptability of psychosocial intervention delivered by non-specialist care providers to the management of children with intellectual disability and ASD [4]. These findings are particularly important in low- and middle-income countries (LMICs) because of the serious shortage of specialists to deliver appropriate intervention services in the population of affected children living in these countries [5]–[7]. While pharmacological treatments, simpler to implement than behavioural interventions, are used for treating challenging maladaptive behavioural problems such as aggression and self-injurious behaviour, as well as co-existing psychiatric or medical conditions, medications do not correct core features of ASD [8]. The primary treatment option in ASD is individualized psychosocial interventions that encompass educational, socialization, behavioural, communication, play leisure and adaptive skill trainings targeted at core areas of autistic impairments [9]. According to the evidence identified by Reichow and colleagues, behavioural analytic techniques delivered by non-specialist providers in community settings, including family members, are effective in addressing these impairments. Interventions provided by non-specialist care providers could help alleviate the scarcity of specialist care by task shifting [10],[11], and potentially also help reduce the risk of burn-out among existing specialists [12],[13].

## Psychosocial Interventions for Children with ASD in LMICs: Challenges to Consider

However, several challenges exist in implementing non-specialist care for children with NDDs. First, non-specialists delivering specialized psychosocial intervention need adequate training and expert supervision [11], and thus cannot replace the need for specialists altogether. Second, care of individuals with NDDs needs to be integrated into the primary health care system and other vital sectors such as schools and welfare [14]. Third, financial resources and technical support are lacking, and little of the research identified by Reichow and colleagues has been conducted in LMICs to help determine how best to surmount these challenges.

## Scarcity of Specialist Services for Children with ASD in LMICs: The Way Forward

Implementation of non-specialist care would benefit from a database of research evidence from LMICs including existing sustainable facilities, available effective interventions, levels of awareness, and the adaptability of the concept of task-shifting and the cultural relevance of specific psychosocial interventions. Adequate resources must be devoted to sustainable training and retraining of non-specialists and financial and technical support for implementation provided. Ultimately, non-specialist psychosocial interventions for NDDs will require advocacy and government support in LMICs, where mortality is given priority over morbidity and disability.
